# Intake of cardiovascular drugs promote severity of anaphylaxis

**DOI:** 10.1186/2045-7022-5-S3-P4

**Published:** 2015-03-30

**Authors:** Sabine Dölle, Günter Edenharter, Franziska Ruëff, Margitta Worm

**Affiliations:** 1 Department of Dermatology and Allergology, Charité-University Medicine Berlin, Allergy Centre Charité, Berlin, Germany; 2 Edenharter Research, Berlin, Germany; 3 Department of Dermatology and Allergology, Ludwig-Maximilian University, Munich, Germany

## Background

Cofactors may contribute to the elicitation and severity of anaphylaxis in about 30% of anaphylactic reactions. Clinical data from registries can support the identification and risk impact of such cofactors. Besides exercise or alcohol, drugs are known to facilitate anaphylactic reactions. The facilitating effect of cardiovascular drugs to hymenoptera stings is controversially discussed; data on the association of cardiovascular drugs and anaphylaxis due to other elicitors are not available.

## Objective

To assess whether cardiovascular drugs like beta-blockers and angiotensin-converting enzyme (ACE)-inhibitors alter the risk for severe anaphylaxis.

## Methods

Data from the German-speaking anaphylaxis registry were collected from January 2006 to March 2013 and analysed. The impact of beta-blockers and/or ACE-inhibitors on the severity of anaphylaxis was calculated by using a logistic regression model.

## Results

The statistical analysis showed an elevated risk of severe anaphylaxis (grade I/II n=2355 versus grade III/IV n=1686) in patients with beta-blocker or ACE-inhibitor treatment (odds ratios (OR): monotherapy with ACE-inhibitor 1.37 [0.94-1.98], monotherapy with beta-blocker 1.36 [1.09-1.72], *p-value* = 0.008 to patient without contribution of cofactors), which was more pronounced when both drugs were taken (OR: combined therapy with ACE-inhibitor/beta-blocker 1.70 [1.28-2.26], *p-value* <0.001). These findings were more prominent if grade I-III versus very severe reactions (grade IV; OR: drug combination 2.44 [1.31-4.53], p-value = 0.005) were calculated. Adjustment of sex and age reduced the OR, however, the results still indicate clinical relevant effect sizes (OR: drug combination 2.07 [1.04-4.12]). The effects were independent of the type of elicitor (food, drug, insect stings and others) of the anaphylactic reaction.

## Conclusion

Our data show that patients treated with beta-blockers and/or ACE-inhibitors have an increased risk to develop more severe anaphylactic symptoms. Interestingly, both drugs seem to synergistically aggravate the anaphylaxis.

